# Synergistic inhibition of avian leukosis virus subgroup J replication by miRNA-embedded siRNA interference of double-target

**DOI:** 10.1186/s12985-015-0277-5

**Published:** 2015-03-21

**Authors:** Rongrong Wei, Xiaoqian Ma, Guihua Wang, Huijun Guo, Jianzhu Liu, Lingxiao Fan, Ziqiang Cheng

**Affiliations:** College of Veterinary Medicine, Shandong Agricultural University, Tai’an, 271018 China; Xiangya School of Medicine, Changsha, 410013 China

**Keywords:** Avian leukosis subgroup J, MiRNA-embedded siRNA interference, Double targets

## Abstract

**Background:**

The diseases caused by avian leukosis virus subgroup J (ALV-J) has become a serious problem in the poultry. Due to largely ineffective vaccines, new control measures are needed to be developed. RNA interference (RNAi) has been developed a promising measure for antivirus in poultry.

**Methods:**

In this study, miRNA-embedded siRNA interference was designed and used to inhibit ALV-J replication in vitro and in vivo. Each sequence of target siRNA derived from the gag (p15), pol (p32), env (gp85) and LTR (U3) gene of ALV-J was embedded into mouse miR-155 backbone as a pre-miRNA hairpin oligonucleotide sequence. After annealing, they were cloned into pcDNA6.2-GW/EmGFP-miR vector, respectively. For detecting the interference effect, recombinant vectors were introduced into DF-1 cells and day-old SPF chickens that infected with ALV-J.

**Results:**

In vitro, single target interference showed effective inhibition of reducing 74% ~ 85% mRNA of ALV-J. Double targets showed more efficient inhibition of reducing 96% ~ 98% mRNA of ALV-J. In vivo, chicks were inoculated with each recombinant plasmid in peritoneal cavity at day of hatch, and monitored infection status at interval 1 day postinfection for 4 weeks. Delivery of single target or double targets miRNA significantly reduced viremia and pathogenicity caused by ALV-J in vivo, especially the double targets.

**Conclusions:**

These data demonstrated that the miRNA-embedded siRNA interference is an efficient method for inhibition of ALV-J replication, especially double targets.

## Background

Avian leukosis virus subgroup J (ALV-J), an oncogenic retrovirus, isolated in the United Kingdom in 1991, has been spread primarily in meat-type chickens and later in egg-type chickens [1-5]. During the past twenty years, ALV-J has emerged as a serious cause of mortality and suboptimal performance in domestic chickens. Significant economic losses caused by ALV-J infection can be associated with immunosuppression, weight loss and myeloid leukosis formation. The transmission of ALV-J is much higher than other ALV subgroups [6], and some birds show immunological tolerance against the virus, thus making control and eradication much more difficult. Due to vaccines are largely ineffective, new measures against ALV-J are needed to be developed. RNA interference (RNAi) has shown a promising antiviral strategy in poultry [7-10].

RNAi regulate posttranscriptional gene using small RNAs produced through multicomplex machinery to guide suppression of complementary transcripts. In general, short hairpin RNAs (shRNAs) shows more efficient than small interfering RNAs (siRNAs) for transient gene knockdown [11,12]. Simple stem-loop shRNAs transcribed from Pol-III promoters enter the processing machinery at the level of Dicer and can be effective RNAi triggers. Several cases showed successes in interference of ALV replication by using shRNA. Hu et al. [13] showed that siRNAs containing sequences of the gag gene of avian leukosis virus (ALV), when electroporated into chicken embryos, were effective at slowing viral propagation. Chen et al. [14] demonstrated that cells expressing shRNA-mirs targeting the tvb receptor sequence or the viral env (B) sequence significantly inhibited ALV-B replication. Meng et al. [15] also demonstrated that the gag target was shown to effectively suppress the replication of ALV-J by 19.0–77.3%. Though above data showed effective interference virus replication by shRNA, however, their enforced expression can saturate endogenous microRNA (miRNA) pathways and result in severe toxicities [16]. Moreover, Gu et al. [17] revealed that Dicer is imprecise in processing commonly used simple stem-loop designs, which increases the likelihood of aberrant guide- and passenger-strand mediated off-target effects.

MicroRNAs (miRNAs) are endogenous substrates for the RNAi machinery. They are initially expressed as ~80 nt long RNA hairpin located within polymerase II (pol II)-derived transcripts referred to as primary transcripts (pri-miRNAs), which are processed within the nucleus into ~60 nt precursor miRNA (pre-miRNA) hairpins by the RNase III enzyme Drosha and its cofactor DGCR8 [18,19]. The loop is removed by further processing in the cytoplasm by another RNase III enzyme Dicer and its cofactor TRBP, yielding an RNA duplex of ~20 bp flanked by 2-nt 3′ overhangs. The RNA strand is loaded into the RNA-induced silencing complex (RISC) and forms the mature miRNA [20,21]. The miRNA then guides RISC to mRNAs bearing complementary target sites [22].

Base on endogenous miRNA biogenesis pathways, a novel approach established with introducing synthetic shRNA stems into the context of endogenous miRNAs can trigger potent knockdown. A number of miRNA backbones can be used, including miR-155, miR-30 and miR17-92 [23-25].

In this study, we screened the optimal sequence of target siRNA in gag, pol, env and LTR of ALV-J genome for RNAi ALV-J replication. The siRNA was embedded into mouse miR-155 backbone to form pre-miRNA hairpin structure, and then constructed into pcDNA6.2-GW/EmGFP-miR vector. After transfection assay, ALV-J infected DF-1 cells were transfected or day old SPF chicks were inoculated with single target or double targets recombinant vectors. Quantitative real time PCR (qPCR), western blot and immunofluorescence assays (IFA) were used to detect interference effect in vitro, and infection status and pathogenicity were evaluated for interference effect in vivo.

## Results

### Construction of expression vectors and its transfection efficiency

**Figure 1:**
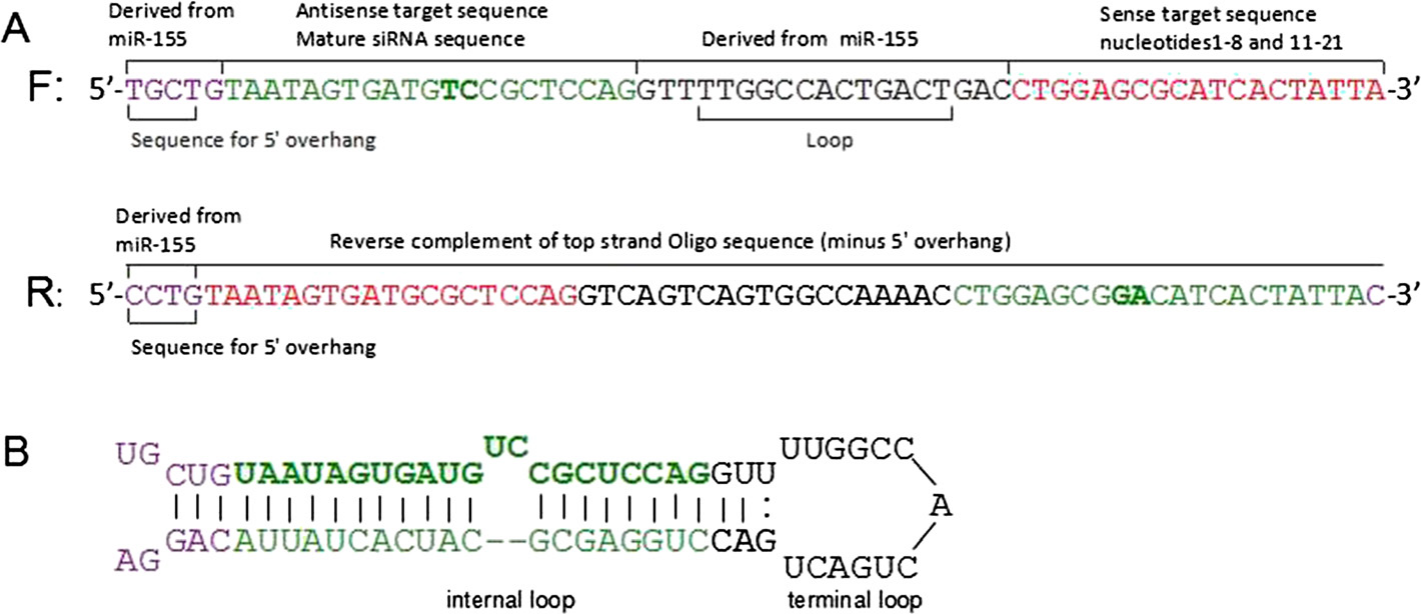
**The forward and reverse sequence of pre**-**miRNA containing target siRNA (A) and the hairpin structures of miRNA**-**embedded siRNA (B).**

**Figure 2:**
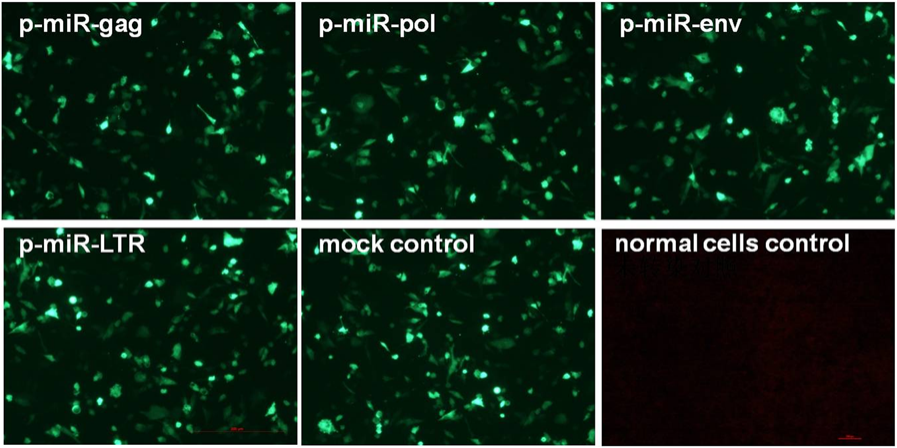
**Transfection effect of recombinant expression vectors observed by fluorescence microscope.** The miRNA-embedded siRNA expression plasmids were transfected into DF-1 cells and GFP expression was monitored at 48 h post-transfection by fluorescence microscopy (magnification 40×).

The synthesis of four pre-miRNA (schematic diagram was shown in Figure 1) and a control sequence was cloned into pcDNA6.2-GW/EmGFP-miR, respectively, and named p-miR-gag, p-miR-pol, p-miR-env, p-miR-LTR and null vector control. Sequence analysis and recombinant plasmid electrophoresis results confirmed that miRNA-embedded siRNA expression vectors were successfully constructed. At 48 h post transfection, typical green fluorescence-positive cells were observed by fluorescence microscopy. As shown in Figure 2, about 70% of the DF-1 cells are positive, indicating the transfection efficiencies is suitable for RNA interference experiment.

### MiRNA-embedded siRNA interference of single target and double targets significantly inhibit ALV-J replication in vitro

To assess the corresponding effects on ALV-J replication, we used each recombinant expression vector to transfect DF-1 cells that infected NX0101 strain of ALV-J. To assess the co-interference effect on ALV-J replication, we used non-coding sequence-LTR combining with other three construct gene (gag, pol and env) to set up three combined groups (gag + LTR, pol + LTR and env + LTR). The combined plasmids were transfected into DF-1 cells that infected with ALV-J. At 72 h post-transfection, the effect of the anti-ALV-J vectors on viral infection was measured by immunofluorescence, western blot and qPCR.

**Figure 3:**
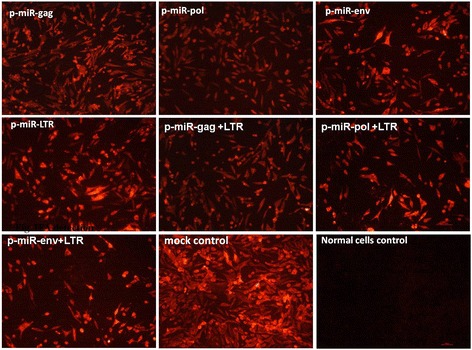
**Single target and double target interference efficiency detected by IFA.** The 200 ng single target miRNA-embedded siRNA expression plasmids and p-miR-LTR (100 ng) combination with p-miR-gag (100 ng), p-miR-pol (100 ng) and p-miR-env (100 ng) were transfected with ALV-J into DF-1 cells and gp85 protein of ALV-J expression was monitored 72 h post-transfection by fluorescence microscopy (magnification 40×). The primary antibody is anti-gp85 monoclonal antibody (1D4), and the second antibody is PE-labeled anti-mouse antibody.

**Figure 4:**
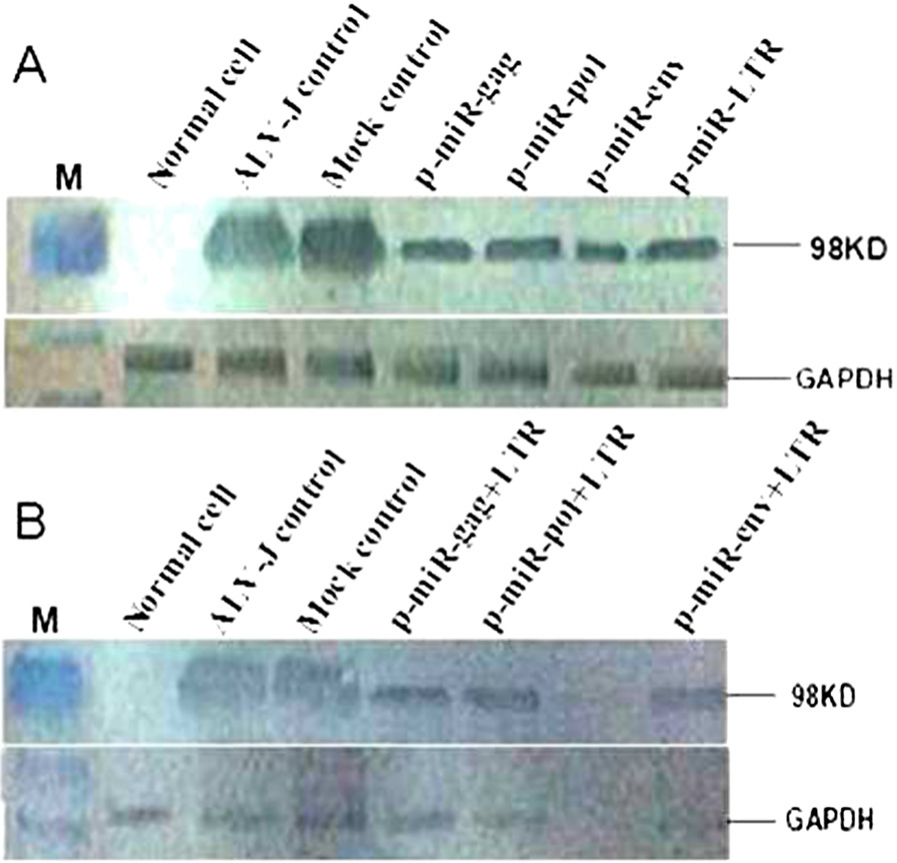
**Western blot detection of ALV**-**J expression. (A)** Single target RNAi; **(B)** double targets RNAi.

As shown in Figures 3 and 4, IFA and western blot results indicated that four recombinant plasmids were all significantly reduced fluorescence intensity compared with ALV-J control, indicating that the significant inhibition of the expression of the ALV-J target gene. All combined recombinant plasmids were significantly reduced expression of ALV-J compared with null control and ALV-J control. No significant differences were detected among the ALV-J control and null vector control.

**Figure 5:**
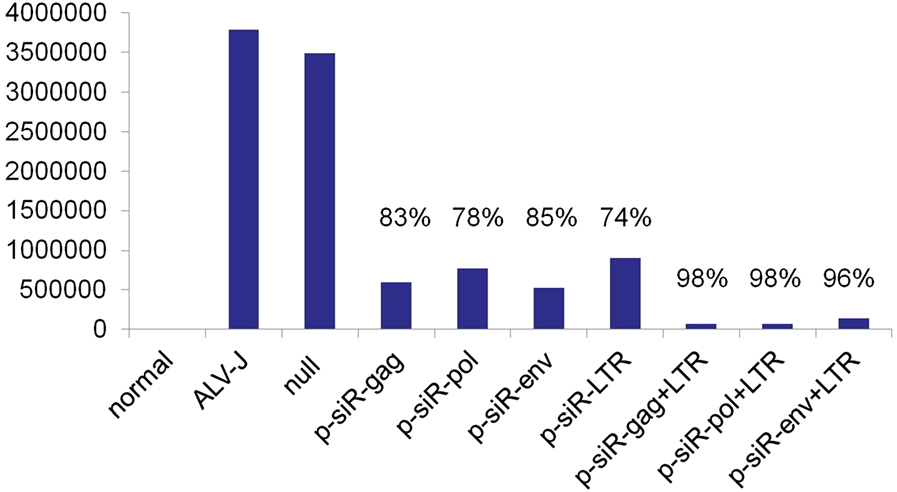
**qPCR detection of single target and double target interference efficiency.** All four single recombinant vectors, p-miR-gag, p-miR-pol, p-miR-env and p-miR-LTR, induced a significant reduction in viral RNA copies of 83%, 78%, 85%, 74%, respectively. Combining p-siR-LTR with p-siR-gag, p-siR-pol and p-siR-env showed more significant reduction in viral RNA copies of 98%, 98% and 96%. Single-target and double-target showed significantly difference (P < 0.05).

The qPCR analysis for env gene expression of ALV-J showed that mRNA levels in cells transfected with four single-targets and three double-targets differed significantly from that observed in the ALV-J control and null vector control groups (P < 0.05) (Figure 5). All four recombinant vectors, p-miR-gag, p-miR-pol, p-miR-env and p-miR-LTR, induced a considerable reduction in viral mRNA copies of 83%, 78%, 85% and 74%, respectively. The interference efficiency of three double-targets for mRNA level of ALV-J is 98%, 98%, and 96%, respectively. Single-targets and double-targets showed significantly difference (P < 0.05). No significant differences were detected among the ALV-J control and null vector control.

### MiRNA-embedded siRNA interference significantly reduce viremia and pathogenicity caused by ALV-J

The anti-virus efficacies of the miRNA-embedded siRNA interference were determined by infection status tested by ELISA for ALV-J antigen and antibody (viremia), leukocyte count, and histopathology observation (pathogenicity) in chickens experimentally inoculated with ALV-J and recombinant plasmids.

Shedding virus result of all groups was shown in Table 1. ALV-J control and null control was continuous shedding virus from day 2. Single target interference group showed no shedding until 8 days for pol target, 10 days for LTR target, 12 days for gag tatget, and 16 days for env target post infection of ALV-J. Consistent with in vitro result, single target of env was found to be the most effective plasmid that postpones the time of shedding virus to 16 days compared to other single target interference groups. Double-target groups showed more protective efficiency compared to single target groups. They can postpone the time of shedding virus from 16 to 22 days. Though shedding virus in interference groups at last, the level of shedding virus was significant lower (p < 0.05) than positive control group, indicating that all interference plasmid can effectively reduce the levels of ALV-J in vivo.

**Table 1:** **p27 antigen of ALV**-**J shedding tested by ELISA**

	**2d**	**4d**	**6d**	**8d**	**10d**	**12d**	**16d**	**20d**	**22d**	**24d**	**28d**
normal control	0.04 ± 0.08	0.04 ± 0.17	0.02 ± 0.06	0.11 ± 0.10	0.03 ± 0.10	0.01 ± 0.06	0.02 ± 0.07	0.01 ± 0.01	0.07 ± 0.05	0.04 ± 0.04	0.07 ± 0.04
null control	0.26 ± 0.02	1.08 ± 0.08	0.46 ± 0.22	1.14 ± 0.21	1.25 ± 0.55	0.43 ± 0.06	1.73 ± 0.76	0.44 ± 0.21	0.86 ± 0.21	0.47 ± 0.24	1.33 ± 0.19
ALV-J control	0.57 ± 0.08	1.23 ± 0.05	0.99 ± 0.37	0.29 ± 0.12	1.43 ± 0.24	0.24 ± 0.09	1.12 ± 0.18	0.45 ± 0.37	1.26 ± 0.08	0.24 ± 0.11	1.36 ± 0.30
p-miR-gag + LTR	0.06 ± 0.08	0.09 ± 0.10	0.08 ± 0.12	0.05 ± 0.06	0.03 ± 0.07	0.08 ± 0.07	0.26 ± 0.10^a^	0.03 ± 0.07	0.05 ± 0.03	0.31 ± 0.17	0.43 ± 0.15
p-miR-pol + LTR	0.03 ± 0.01	0.02 ± 0.00	0.02 ± 0.00	0.03 ± 0.00	0.01 ± 0.00	0.04 ± 0.02	0.03 ± 0.08	0.02 ± 0.05	0.21 ± 0.08^a^	0.38 ± 0.06	0.19 ± 0.10
p-miR-env + LTR	0.03 ± 0.00	0.02 ± 0.00	0.02 ± 0.01	0.13 ± 0.00	0.02 ± 0.00	0.04 ± 0.03	0.14 ± 0.01	0.02 ± 0.02	0.33 ± 0.04^a^	0.37 ± 0.17	0.08 ± 0.13
p-miR-gag	0.04 ± 0.00	0.02 ± 0.00	0.06 ± 0.00	0.08 ± 0.01	0.17 ± 0.00	0.31 ± 0.04^a^	0.02 ± 0.07	0.22 ± 0.06	0.3 ± 0.08	0.02 ± 0.06	0.38 ± 0.23
p-miR-pol	0.05 ± 0.01	0.08 ± 0.01	0.12 ± 0.00	0.20 ± 0.00^a^	0.25 ± 0.01	0.02 ± 0.03	0.09 ± 0.01	0.01 ± 0.05	0.31 ± 0.13	0.22 ± 0.14	0.43 ± 0.15
p-miR-env	0.01 ± 0.01	0.01 ± 0.01	0.02 ± 0.00	0.01 ± 0.01	0.02 ± 0.00	0.15 ± 0.11	0.22 ± 0.06^a^	0.01 ± 0.05	0.01 ± 0.15	0.04 ± 0.05	0.32 ± 0.07
p-miR-LTR	0.04 ± 0.01	0.03 ± 0.01	0.04 ± 0.04	0.18 ± 0.01	0.29 ± 0.00^a^	0.22 ± 0.03	0.01 ± 0.06	0.11 ± 0.21	0.44 ± 0.06	0.32 ± 0.18	0.09 ± 0.11

Note: ^a^indicate the first time of shedding p27 antigen of ALV-J. Cut off value = 0.2.

Histopathological analysis at 14 days post infection showed that obvious foci of inflammatory filtration was present in various tissues of ALV-J infected and null control group, while no any invisible lesions was observed in RNAi groups. At 28 days post infection, in ALV-J and null control group, more serious lesions were developed, while in RNAi group, only mild inflammatory filtration was observed (data not shown).

Overall, viremia or pathogenicity in both the single and double target groups were postponed or reduced compared to the positive control groups, suggesting that the expression of anti-ALV-J miRNA-embedded siRNA was able to decrease replication of pathogenic ALV-J in chickens.

## Discussion

The power of RNAi to silence specific genes is now widely used in the laboratory to explore the functions of genes. RNAi generally arises from two types of intermediary molecules: micro-RNAs (miRNAs) and small interfering RNAs (siRNAs). Both siRNA and miRNA can stop production of specific proteins by triggering degradation of specific mRNAs. However, due to its simple short-hairpin, the interference efficiency of siRNA is less than miRNA. miRNA genes transcribed as larger “pri-miRNA” precursors have been found to more effectively generate an RNAi effect. In this case, we designed to replace the stem region of a miRNA gene with the target viral siRNA sequence and its guide RNA complement, namely, constructing a pri-miRNA containing target viral siRNA, and then to test the interference efficiency. The results demonstrated that the method of miRNA embedded siRNA is widely effective for any genes in ALV-J at inhibiting viral replication, and the interference efficiency is higher than previous report regardless of any single target. For getting higher interference efficiency, we used double targets to co-transfect DF-1 cells that infected by ALV-J. Since ALV-J comprise of three constructive genes of gag, pol and env, and two non-coding genes of LTR. So, we designed LTR combining with other three constructive genes. The RNAi result showed that the double-target RNAi dramatically decreased ALV-J mRNA copies in cultured cells, suggesting that double target of miRNA embedded siRNA is more efficient method for inhibition of ALV-J replication and possible for application of clinical practice.

Comparing simple stem-loop, the pre-miRNA (miRNA-embedded siRNA) which has double loops has several advantages: (1) like endogenous miRNAs, miRNA-embedded siRNA can be expressed more efficiently and higher interference efficiency [18,19]; (2) multiple miRNA-embedded siRNA can be expressed as a polycistron, providing a setup for combinatorial RNAi studies [23,26]; (3) miRNA-embedded siRNA are less prone to cause toxicities by interfering with endogenous miRNA pathways [27]; (4) the natural loop configuration of miRNA-embedded siRNA ensures precise Dicer cleavage and reduces off-target effects [17].

Furthermore, a major concern for the miRNA embedded siRNA is the possibility of clinical application. So, we singlely or doubly delivered plasmids into body cavity of day-old chicks by injection. For getting obvious results, we took high dose of 10^5.2^ TCID_50_ ALV-J and Lipofectamin 2000 coated recombinant plasmids. The results demonstrated that the plasmid containing miRNA embedded siRNA can significantly reduce ALV-J viremia and pathogenicity, especially in double-target RNAi groups, although it could not completely inhibit ALV-J replication. The incomplete inhibition could be due to the high dose of ALV-J, the administration route of recombinant plasmids, or the vectors and so on. The apparent higher titer of the ALV-J challenge and improper administration route may have impaired the interference ability of recombinant plasmid. For clinical practice, the optimal RNAi condition in vivo is needed to be further studied in future.

## Conclusions

This study has demonstrated that miRNA embedded siRNA method is an effective method to inhibit ALV-J replication in vitro and in vivo. The double-target of miRNA-embedded siRNA showed more powerful inhibitive effect than those of single target. The RNAi of miRNA embedded siRNA has a potential value for clinical application in poultry flocks.

## Materials and methods

### Virus, cells and animals

The animals used in this study were approved by the Shandong Animal Care and Use Committee (SDACUC number 14-095). The stock virus, NX0101 stain of ALV-J, was isolated from in broiler breeder by our lab. The virus titer was determined by limiting dilution in DF-1 culture. Briefly, DF-1 cells were seeded at 5 × 10^5^ cells/well of 6-well plate and infected the following day with the NX0101 strain of ALV-J. Culture supernatants and cells were collected at different time point postinfection and stored at -80°C. Growth curve of the virus was monitored by qPCR. DF-1 cell line, a continuous line of chicken embryo fibroblasts, is derived from an EV-0 embryo and is free of endogenous sequences related to the avian sarcoma and leukosis virus (ALSV) group [28]. All DF-1 cells were maintained in DMEM supplemented with10% FBS. Day old of Leghorn SPF chickens (Saisi) were used for in vivo RNAi experiment.

### Design and production of pre-miRNA

The nucleotide sequences homology of NX0101 (subgroup J, DQ115805) with MQNCSU (subgroup A, DQ365814), SDAU09E3 (subgroup B, JF826241), RSV-Prague (subgroup C, J02342), RSV-Schmidt-Ruppin D (subgroup D, D10652), SD0501(subgroup E, EM467236), HPRS103 (subgroup J, Z46390) and ADOL-7501 (subgroup J, AY027920) that obtained from the National Center for Biotechnology Information (NCBI) were analyzed using Clustal W Method of the DNA Star software, selecting the highest homology target sequences. Furthermore, using software available at http://rnaidesigner.lifetechnologies.com/rnaiexpress, four RNA oligonucleotide sequences, gag (p15)-2477, pol (p32)-4616, env (gp85)-6146 and LTR(U3)-7497, were selected as target siRNA based on the homology information of above ALV subgroup (Table 2). Each pre-miRNA sequence (Table 3) was designed as a single-stranded DNA oligonucleotide, ~64 nt in length and encoding, in order: sequence for 5′ overhang, a 21 nt target antisense siRNA sequence; a mouse miR-155 loop; and the 19 nt sequence deleted nucleotide No 9 and No10 of target sense siRNA sequence, with a corresponding complementary single-stranded DNA oligonucleotides. All pre-miNRA sequences were synthesized by Invitrogen.

**Table 2:** **Sequence of target siRNA**

**Target gene**	**siRNA sequence**	**Start position**
gag-p15	CTGGAGCGGACATCACTATTA	2477
pol-p32	ACAGATATGGCAGACAGACTT	4616
env-gp85	GTACAGCGGAATGGAATTATT	6146
LTR-U3	AGGCAACAGACGGGTCTTATA	7497
Null control	GTCTCCACGCGCAGTACATTT	/

**Table 3:** **Sequence of pre**-**miRNA of miRNA**-**embeded siRNA**

**Oligo name**	**oligo DNA sequences 5′-** **3′**
Gag-p15-top	tgctgTAATAGTGATGTCCGCTCCAGGTTTTGGCCACTGACTGACCTGGAGCGCATCACTATTA
Gag-p15-bottom	cctgTAATAGTGATGCGCTCCAGGTCAGTCAGTGGCCAAAACCTGGAGCGGACATCACTATTAc
Pol-p32-top	tgctgAAGTCTGTCTGCCATATCTGTGTTTTGGCCACTGACTGACACAGATATCAGACAGACTT
Pol-p32- bottom	cctgAAGTCTGTCTGATATCTGTGTCAGTCAGTGGCCAAAACACAGATATGGCAGACAGACTTc
Env-gp85-top	tgctgAATAATTCCATTCCGCTGTACGTTTTGGCCACTGACTGACGTACAGCGATGGAATTATT
Env-gp85- bottom	cctgAATAATTCCATCGCTGTACGTCAGTCAGTGGCCAAAACGTACAGCGGAATGGAATTATTc
LTR-U3-top	tgctgTATAAGACCCGTCTGTTGCCTGTTTTGGCCACTGACTGACAGGCAACACGGGTCTTATA
LTR-U3- bottom	cctgTATAAGACCCGTGTTGCCTGTCAGTCAGTGGCCAAAACAGGCAACAGACGGGTCTTATAc
Null-top	tgctgAAATGTACTGCGCGTGGAGACGTTTTGGCCACTGACTGACGTCTCCACGCAGTACATTT
Null- bottom	cctgAAATGTACTGCGTGGAGACGTCAGTCAGTGGCCAAAACGTCTCCACGCGCAGTACATTTc

### Construction of miRNA-embedded siRNA expression vectors

Two complementary oligonucleotides were denatured and annealed into double-stranded DNAs, and then were inserted into a linearized expression vector, pcDNA6.2-GW/EmGFP-miR (Invitrogen). The pcDNA6.2-GW/EmGFP-miR vectors had the BLOCK-iT Lentiviral Pol II miR RNAi Expression Systems that were designed to express artificial pre-miRNA hairpin which were excised by Drosha when present in the nucleus. After excision, the pre-miRNA hairpin is exported to the cytoplasm for further processing by Dicer. One strand of the resulting miRNA duplex intermediate is incorporated into RISC, where it acts as a guide RNA to target RISC to fully complementary ALV-J mRNAs. This primarily results in translational inhibition and mRNA degradation. The expressed structure of miRNA-embedded siRNA is shown in Figure 1B. All recombinant plasmids were sequenced (F:5′- GGCATGGACGAGCTGTACAA -3′; R: 5′- CTCTAGATCAACCACTTTGT -3′; invitrogen) to confirm the inserted sequences are right. Each recombinant plasmid was transformed into competent cell DH5α and smeared on LB solid medium containing 50 μg/ml of spectinomycin dihydrochloride (Sigma) for amplification. The positive clones were propagated and cultured in liquid LB. The recombinant plasmids were extracted with endotoxin-free plasmid extraction kit (Sigma).

### Recombinant vector transfection

DF-1 cells were seeded in 24-well plates (70% confluence) and transfected with recombinant vectors (200 ng/well) by using Lipofectamin 2000 (Invitrogen) according to the manufacturer’s instructions. This concentration was determined to be optimal in preliminary experimentation. At 48 h post transfection, GFP expression from the vectors was detected by fluorescence microscopy as described previous [14].

### MiRNA-embedded siRNA inhibition assay for ALV-J replication in vitro

Single target and double targets interference strategy were used in vitro study. When DF-1 cells that infected with ALV-J 10^2^ TCID_50_ had grown to 80% confluence, they were transfected with the RNAi recombinant expression plasmid (single target, 200 ng/well or double targets, 100 ng/per plasmid/well) using X-treme GENE HP DNA Transfection Reagent. At 6 h post transfection, culture media was aspirated and replenished with DMEM containing serum and 2% peracetic acid (PAA). At 48 h post transfection, transfection efficiency was determined by the expression of green fluorescent protein (GFP) observed under a fluorescence microscope. DF-1 cells were harvested at 72 h post transfection, and the inhibitory effect was determined by IFA, qPCR and western blot. The negative control and null vector control group were simultaneously analyzed.

### MiRNA-embedded siRNA anti-ALV-J infection in vivo

Day-old of Leghorn SPF chicks were randomly divided into ten groups, 10 chicks each group. Chicks were inouculated with 100 μL 10^5.2^ TCID_50_ ALV-J and recombinant plasmid (2.5 mg/per chick for single target; 1.25 + 1.25 mg/per chick for double-target) with Lipofectamin TM2000 (Invitrogen) in peritoneal cavity, and were breed for four weeks (Table 4). Infection status was identified by ELISA for antibody and antigen test at interval day. Briefly, serum samples from jugular vein were tested for the presence of ALV-J antibodies and cloaca swabs were tested for the presence of group specific antigen (p27). Viral loads in blood were detected by quantitative real time RT-PCR (qPCR). Chickens were necropsied at the termination of the experiment, and the gross and histopathology lesions were observed.

**Table 4:** **Virus or vector inoculation dose of each group in vivo RNAi experiment**

**Group**	**Number of chickens**	**ALV**-**J dose (μL 10** ^**5.2**^ **TCID**_**50**_ **)**	**Vector dose (ng)**
Normal control	10	-	-
Null control	10	100	2500
ALV-J control	10	100	-
p-miR-gag + p-miR-LTR	10	100	1250 + 1250
p-miR-pol + p-miR-LTR	10	100	1250 + 1250
p-miR-env + p-miR-LTR	10	100	1250 + 1250
p-miR-gag	10	100	2500
p-miR-pol	10	100	2500
p-miR-env	10	100	2500
p-miR-LTR	10	100	2500

### Immunofluorescence assays (IFA)

DF-1 cells that infected by ALV-J were transfected with recombinant plasmid using the methods described above. At 72 h post transfection, cells were fixed with ice-cold 40% ethanol and 60% acetone and incubated with the 1D4 monoclonal antibody against ALV-J (dilution, 1:1000) for 1 h at 37°C. The cells were washed three times (10 min per wash) with phosphate-buffered saline (PBS) and incubated with the secondary antibody, PE-labeled goat anti-mouse IgG for 1 h at 37°C. After three washes with PBS, the cells were covered with 50% glycerin and examined under a fluorescence microscope.

### Quantitative real time RT-PCR (qPCR) assay for viral mRNA

RNA of DF-1 was collected at 72 h and treated with DNase I using the RNeasy kit according to manufacturer’s instructions (Qiagen). cDNA was prepared by PrimeScript Kit (TAKARA). Amplifications of target gene of gp85 and chicken GAPDH sequences were carried out in separate reactions using the primers as showed in Table 5. PCR was done in 50 μL containing 25 μL 2× PCR master mix (Promega), 2 μL cDNA, and primers at 2.5 pmol each for 5 min at 95°C followed by 35 cycles of 30s at 95°C, 30 s at 55°C and 30 s at 72°C. The predict segments are 144 bp and 132 bp. The amplified products were cloned into pMD18-T vector (Promega), and plasmid DNA was expanded, purified and accurately quantified by UV-light spectroscopy. Serial dilutions of the plasmid were prepared (from 10^6^ to 10^−1^ copies per reaction) and used to build a standard curve for quantification.

**Table 5:** **Primers used to amplify ALV**-**J genes**

**Target gene**	**Primer sequence**	**Product size(bp)**
Env	Forward TGCGTGCGTGGTTATTATTTC	144
	Reverse AATGGTGAGGTCGCTGACTGT	
GADPH	Forward GAACATCATCCCAGCGTCCA	132
	Reverse CGGCAGGTCAGGTCAACAAC	

qPCR was performed using reagents from the SYBR Premix Ex Taq Kit (ROCHE). Amplification of target gene or reference gene was done in 20 μL volume, containing 10.4 μL SYBR Ex Taq (2×, containing ROX), 0.2 μL P1 (10 mmol/L), 0.2 μL P2 (10 mmol/L), 2 μL cDNA; and 10.4 μL SYBR Ex Taq (2×, containing ROX), 0.4 μL S1(10 mmol/L), 0.4 μL S2(10 mmol/L), 2 μL cDNA, respectively. Run in qPCR instrument using two-step protocol: 95°C for 30 sec. 43 cycles were done with denaturation at 95°C, for 5 sec, annealing at 60°C for 34 sec, and extension at 72°C for 13 sec. Check melt curve profiles for fluorescent nucleic acid binding dye detection and ensure that products are specific.

qPCR data were analyzed using the comparative Ct method(△△Ct) [29]. Differences between the Ct values of the target gene (env) and the internal control (△Ct = Ct target-Ct internal control) were calculated to normalize the differences in the amount of total cDNA added to each reaction and the efficiency of the qPCR. The negative control was used as a reference for each comparison. Differences between the △CT of each env expression plasmid and reference sample (△△Ct = (Ct target-Ct internal control) env plasmid-(Ct target-Ct internal control)NC) were calculated. The expression level of the target gene could be calculated by 2-△△Ct and the value stood for an n-fold difference relative to the negative sample. Results are presented as the mean ± standard deviation(s).The *T* test was performed using SPSS 13.0 statistical software. A P value less than 0.05 was considered statistically significant.

### Western blot

Transfected DF-1 cells were washed with phosphate-buffered saline and then lysed in 2× Laemmli loading buffer (Bio-RAD). Proteins were boiled for 5 min, and then were separated by SDS-polyacrylamide gel electrophoresis (PAGE). After SDS-PAGE, the proteins were transferred onto nitrocellulose membrane. The membrane was stained with 2.5% fast green in 10% acetic acid for 2 min to visualize blotted proteins. Destaining was performed with 10% acetic acid for 10 min. The membrane was blocked with 10% skim milk. The ALV-J SU protein was detected using the 1D4 monoclonal antibody against ALV-J (dilution, 1:1000). For detection of glyceraldehyde-3-phosphate dehydrogenase (GAPDH), a monoclonal antibody (Ambion) was used at a dilution of 1:4,000. Peroxidase-coupled secondary antibodies directed against mouse immunoglobulin (Pierce) were diluted 1:10,000.
